# A Streamlined Approach to Antibody Novel Germline Allele Prediction and Validation

**DOI:** 10.3389/fimmu.2017.01072

**Published:** 2017-09-04

**Authors:** Ben S. Wendel, Chenfeng He, Peter D. Crompton, Susan K. Pierce, Ning Jiang

**Affiliations:** ^1^McKetta Department of Chemical Engineering, Cockrell School of Engineering, The University of Texas at Austin, Austin, TX, United States; ^2^Department of Biomedical Engineering, Cockrell School of Engineering, The University of Texas at Austin, Austin, TX, United States; ^3^Laboratory of Immunogenetics, National Institute of Allergy and Infectious Diseases, National Institutes of Health, Rockville, MD, United States; ^4^College of Natural Sciences, Institute for Cellular and Molecular Biology, The University of Texas at Austin, Austin, TX, United States

**Keywords:** antibody, B cell, immune repertoire sequencing, *IGHV*, novel germline allele, polymorphism

## Abstract

Advancements in high-throughput sequencing and molecular identifier-based error correction have opened the door to antibody repertoire sequencing with single mutation precision, increasing both the breadth and depth of immune response characterization. However, improvements in sequencing technology cannot resolve one key aspect of antibody repertoire sequencing accuracy: the possibility of undocumented novel germline alleles. Somatic hypermutation (SHM) calling requires a reference germline sequence, and the antibody variable region gene alleles collected by the IMGT database, although large in number, are not comprehensive. Mismatches, resulted from single nucleotide polymorphisms or other genetic variation, between the true germline sequence and the closest IMGT allele can inflate SHM counts, leading to inaccurate antibody repertoire analysis. Here, we developed a streamlined approach to novel allele prediction and validation using bulk PBMC antibody repertoire sequencing data and targeted genomic DNA amplification and sequencing using PBMCs from only 4 ml of blood to quickly and effectively improve the fidelity of antibody repertoire analysis. This approach establishes a framework for comprehensively annotating novel alleles using a small amount of blood sample, which is extremely useful in studying young children’s immune systems.

## Introduction

V(D)J recombination and non-template nucleotide insertion in the junction regions generate the first level of antibody repertoire diversity. During an immune response, B cells that are activated by binding their matching antigens go through a clonal expansion process accompanied by somatic hypermutations (SHMs) that are quasi-randomly introduced to the antibody genes. These mutated antibodies are then selected based on binding strength to the antigen, leading to a second generation of higher affinity antibodies (1–3).

Antibody repertoire SHM patterns have been implicated in a wide range of applications, from the development of broadly neutralizing antibodies against HIV to the diminished effectiveness of vaccines in elderly subjects (4–6). The recent incorporation of molecular barcodes into high-throughput immune repertoire sequencing has improved the ability to discern individual SHMs from PCR and sequencing errors (7, 8); however, accurate SHM calling requires an accurate set of reference germline sequences to align to. The polygenic and polyallelic nature of the variable domain locus confounds this issue. Currently, 259 functional human antibody heavy chain V gene alleles listed in the IMGT database can be broken into seven subfamilies that likely share common evolutionary ancestors based on sequence similarity (9), but recent studies have shown that individuals often carry novel alleles that have yet to be characterized in the IMGT database (10–12). These novel alleles can be problematic for antibody repertoire analysis because single nucleotide polymorphisms (SNPs) between the novel alleles and the nearest IMGT alleles will instead be counted as SHMs on every sequence utilizing that allele, inflating the SHM load and skewing the SHM patterns. Although there are several software tools (11, 12) to predict the existence of novel alleles, a simple method for novel allele prediction and validation is lacking, especially using a small amount of blood samples.

Here, we report a streamlined method for predicting novel alleles from bulk antibody repertoire data and validating them by sequencing unrecombined genomic DNA (gDNA) from non-B cells. This method can be applied to PBMCs and B cells purified from as little as 4 ml of blood. Six novel alleles across eight different subjects from a larger, ongoing malaria study cohort (13) were predicted and validated with perfect congruency between the expressed repertoire and gDNA. This method can quickly and easily be applied to any antibody repertoire data to mitigate the effects of germline mismatches on SHM patterns.

## Materials and Methods

### Study Design and Cohort

PBMC samples from eight residents of Kalifabougou, Mali were collected from an ongoing malaria cohort study (13). Up to five million PBMCs were directly lysed for antibody repertoire sequencing, and T cells were FACS-sorted from the remaining PBMCs for unrecombined gDNA validation. 6 predicted novel alleles were chosen for validation. The Ethics Committee of the Faculty of Medicine, Pharmacy, and Dentistry at the University of Sciences, Technique, and Technology of Bamako and the Institutional Review Board of the National Institute of Allergy and Infectious Diseases, National Institutes of Health approved the malaria study, from which we obtained frozen PBMCs. Written informed consent was obtained from adult participants and from the parents or guardians of participating children. The study is registered in the www.ClinicalTrials.gov database (NCT01322581).

### Antibody Repertoire Sequencing and Novel Allele Prediction

**Figure 1:**
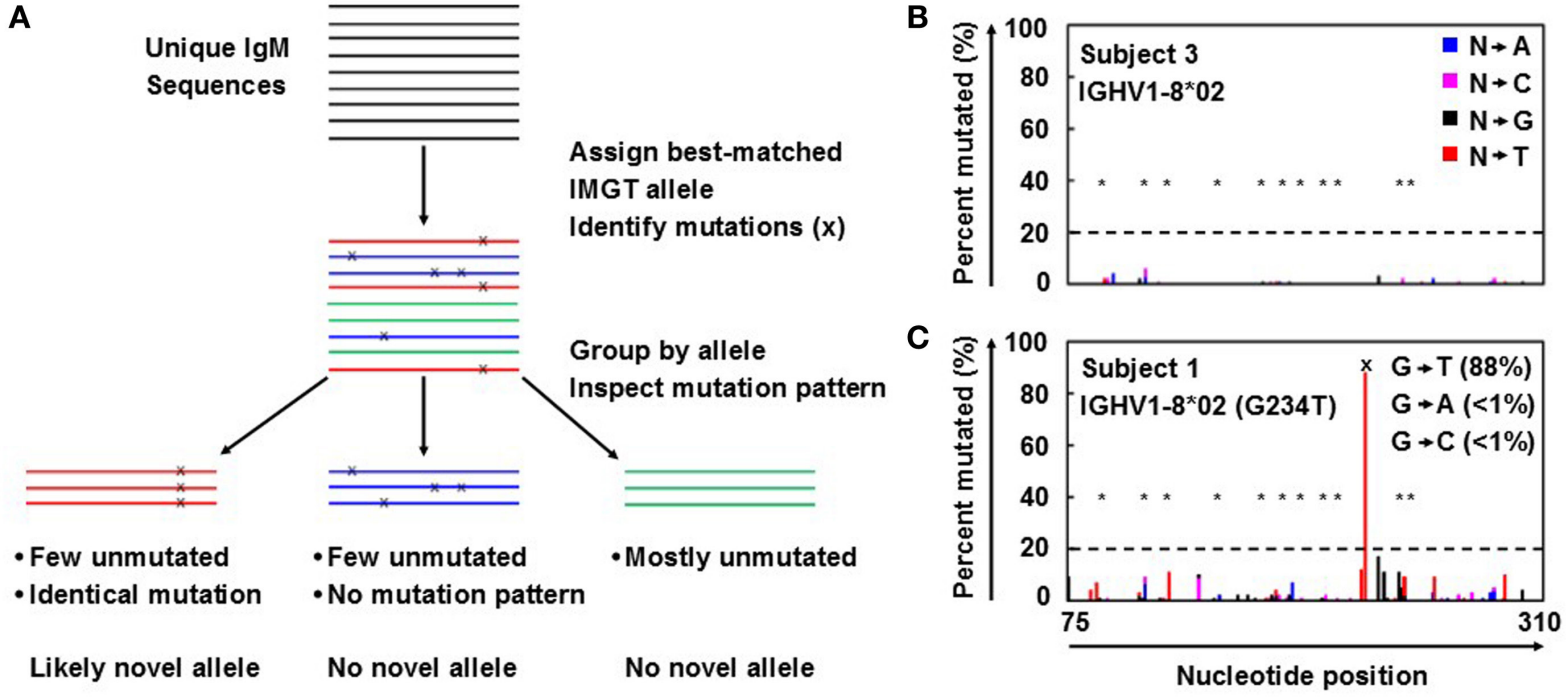
Novel germline allele prediction from bulk repertoire sequencing data. **(A)** Overview of novel allele prediction schematic from bulk repertoire sequencing data. Color indicates best-matched IMGT reference germline allele assignment; x indicates single nucleotide polymorphisms (SNP) to reference germline allele. **(B,C)** Representative percent of unique IgM sequences mutated for each position along the sequence for the absence **(B)** and presence **(C)** of a novel allele resulted from SNP(s). Color indicates the nucleotide substitution: A (blue), C (pink), G (black), and T (red). *Indicates somatic hypermutation hotspots; dashed line indicates prediction threshold of SNP calling; x indicates SNP on predicted novel allele compared to the closest IMGT reference germline allele. SNP is broken down by nucleotide substitution as indicated in inset in **(C)**.

Antibody repertoire sequencing was performed as previously described (7, 8) with some modifications. Novel allele prediction schematic is summarized in Figure 1A. In short, unique IgM sequences from bulk PBMC samples were used to minimize the effects of SHM and clonal expansion, as they are more likely to be derived from naïve B cells and thus have fewer SHMs than other antibody isotypes. These sequences were first aligned to the reference germline allele database (e.g., IMGT) and assigned to the best-matched alleles. The ratios of perfectly matched sequences to those with 1, 2, 3, and 4 mismatches (putative SNPs) compared to the reference germline were determined. Ratios of less than 2 to 1 were then inspected for identical mutation patterns. If identical mutations were present in at least 20% of the unique sequences, with less than 2% of the sequences harboring different mutations at the same positions, the allele containing those SNPs was flagged as a possible novel allele.

### gDNA Sequencing and Reads Processing

Nested PCR was used to reduce non-specific amplification. Primers were designed such that the inner primers were no fewer than 14 bases away from the locations of the predicted IMGT/novel allele mismatches. Inner primers were fused to partial Illumina adaptors, and a third PCR was performed to add the full adaptor sequence (Table S1 in Supplementary Material). First PCR was performed on 10% of purified gDNA from 2,000 sorted T cells using Phusion Hot Start II DNA Polymerase (Thermo Scientific) with the following protocol: 98°C for 1 min; 10 cycles of 98°C for 30 s, 57°C for 1 min, and 72°C for 5 min; then 72°C for 10 min. Second PCR was performed on 10% of the first PCR product with the same protocol. Final adaptor ligation was performed on 10% of the second PCR product using TaKaRa Ex Taq DNA Polymerase Hot Start with the following protocol: 95°C for 3 min; 10 cycles of 95°C for 30 s, 57°C for 30 s, and 72°C for 2 min; then 72°C for 7 min. Libraries were pooled, gel-purified, and sequenced *via* Miseq 2 × 250 PE.

Sequencing reads were first merged using the SeqPrep tool.^1^ IgBlast (14) was then used to align the reads to the established IMGT germline allele database. Reads mapping to the nearest germline allele to the novel allele of interested were filtered. Reads matching exactly to the IMGT germline allele or the novel allele sequence were tallied. If the exact novel allele sequence was found in 20% or more of the tallied reads, the sample was considered a positive hit.

### Data Availability

Antibody repertoire sequencing data can be found in dbGaP under the accession number phs001209.v1.p1. gDNA sequencing data can be found in SRA under the accession number SRP112759.

## Results

### Bulk Antibody Repertoire Novel Allele Prediction

Antibody repertoire sequencing data from bulk PBMCs were collected and processed as described in Section “Antibody Repertoire Sequencing and Novel Allele Prediction” and summarized in Figure 1A. IgM sequences were used to calculate the mutation distribution by position because IgM is mostly expressed on naïve B cells that have not been activated and have fewer SHMs compared to other isotypes. As expected, the percentage of unique sequences mutated at each position in IgM is low, even for SHM hotspots (Figure 1B). However, a large spike at one (or more) specific position(s) could indicate the presence of a novel allele resulted from SNP(s) (Figure 1C).

A threshold of 20% of unique sequences harboring the identical predicted SNP(s) was applied to determine a positive hit on novel allele (Figures 1B,C, dashed horizontal line). Several *IGHV* genes, e.g., *IGHV1-69* and *IGHV3-30*, have copy-number variants (CNVs) that arose from chromosomal segmental duplication and insertion/deletion events, leading to a diploid copy number ranging from 0 to 4 alleles present for a given gene for an individual (15, 16). For genes with up to 4 copies, this 20% threshold can account for a heterozygous genotype with 1 of 4 copies being the novel allele, which should have a 25:75 split on the usage of these four alleles. Six genes with predicted novel alleles were chosen for validation (Table 1, column headers). Novel alleles were named according to the nearest IMGT allele followed by the substitution(s) in parenthesis, e.g., novel allele IGHV1-8*02 (G234T) has the same sequence as the IMGT allele IGHV1-8*02 with the G at position 234 substituted with a T. The full novel allele sequences can be found in Table 2. These novel alleles were also predicted independently using TIgGER (11), another novel germline allele detection tool. Overall, 17 positive novel allele hits were predicted from the 6 genes across the 8 subjects.

**Table 1:** Summary of genomic DNA (gDNA) validation of novel alleles predicted by bulk repertoire sequencing data.

	Novel alleles					
Subjects	IGHV1-8*02 (G234T)	IGHV1-69*01 (G163A)	IGHV3-30*02 (T201C)	IGHV4-31*02 (C198T)	IGHV4-59*01 (T109C)	IGHV4-61*01 (C93T_C136G_A138C)
1	+/+	+/+	+/+^a^	−/−	+/+	−/−
2	−/N.D.	−/−	−/−	+/+	−/−	−/−^b^
3	−/−	+/+^a^	−/−	−/−	+/+	+/+
4	+/+	−/−	−/−^a^	−/−	−/−	−/−
5	−/−	−/−	−/−	+/+	−/−	−/−
6	+/+	−/−^a^	+/+	−/−^b^	+/+	−/−^b^
7	+/+	−/−	+/+^a^	−/−^b^	−/−	−/−
8	+/+	+/+	−/−	−/−^b^	−/−	−/−

*+/+ (dark green) indicates positive in both the bulk repertoire and the gDNA data for predicted single nucleotide polymorphisms (SNPs); −/− (light green) indicates negative in both the bulk repertoire and the gDNA data for predicted SNPs; −/N.D. (yellow) indicates negative in bulk repertoire data but gDNA failed to amplify during gDNA validation for predicted SNPs*.

*^a^Indicates the existence of copy-number variants with more than two alleles detected in the gDNA data that belong to the same gene*.

*^b^Indicates the gene was not detected in the repertoire or gDNA, possibly due to gene deletion*.

**Table 2:** Novel allele sequences.

Novel allele	Sequence
IGHV1-8*02 (G234T)	CAGGTGCAGCTGGTGCAGTCTGGGGCTGAGGTGAAGAAGCCTGGGGCCTCAGTGAAGGTC TCCTGCAAGGCTTCTGGATACACCTTCACCAGCTATGATATCAACTGGGTGCGACAGGCC ACTGGACAAGGGCTTGAGTGGATGGGATGGATGAACCCTAACAGTGGTAACACAGGCTAT GCACAGAAGTTCCAGGGCAGAGTCACCAT 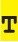 ACCAGGAACACCTCCATAAGCACAGCCTAC ATGGAGCTGAGCAGCCTGAGATCTGAGGACACGGCCGTGTATTACTGTGCGAGAGG
IGHV3-30*02 (T201C)	CAGGTGCAGCTGGTGGAGTCTGGGGGAGGCGTGGTCCAGCCTGGGGGGTCCCTGAGACTC TCCTGTGCAGCGTCTGGATTCACCTTCAGTAGCTATGGCATGCACTGGGTCCGCCAGGCT CCAGGCAAGGGGCTGGAGTGGGTGGCATTTATACGGTATGATGGAAGTAATAAATACTA 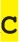 GCAGACTCCGTGAAGGGCCGATTCACCATCTCCAGAGACAATTCCAAGAACACGCTGTAT CTGCAAATGAACAGCCTGAGAGCTGAGGACACGGCTGTGTATTACTGTGCGAAAGA
IGHV4-61*01 (C93T_C136G_A138C)	CAGGTGCAGCTGCAGGAGTCGGGCCCAGGACTGGTGAAGCCTTCGGAGACCCTGTCCCTC ACCTGCACTGTCTCTGGTGGCTCCGTCAG 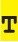 AGTGGTAGTTACTACTGGAGCTGGATCCGG CAGCCC 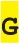 C 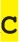 GGGAAGGGACTGGAGTGGATTGGGTATATCTATTACAGTGGGAGCACCAAC TACAACCCCTCCCTCAAGAGTCGAGTCACCATATCAGTAGACACGTCCAAGAACCAGTTC TCCCTGAAGCTGAGCTCTGTGACCGCTGCGGACACGGCCGTGTATTACTGTGCGAGAGA
IGHV4-59*01 (T109C)	CAGGTGCAGCTGCAGGAGTCGGGCCCAGGACTGGTGAAGCCTTCGGAGACCCTGTCCCTC ACCTGCACTGTCTCTGGTGGCTCCATCAGTAGT 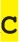 ACTACTGGAGCTGGATCCGGCAGCCC CCAGGGAAGGGACTGGAGTGGATTGGGTATATCTATTACAGTGGGAGCACCAACTACAAC CCCTCCCTCAAGAGTCGAGTCACCATATCAGTAGACACGTCCAAGAACCAGTTCTCCCTG AAGCTGAGCTCTGTGACCGCTGCGGACACGGCCGTGTATTACTGTGCGAGAGA
IGHV1-69*01 (G163A)	CAGGTGCAGCTGGTGCAGTCTGGGGCTGAGGTGAAGAAGCCTGGGTCCTCGGTGAAGGTC TCCTGCAAGGCTTCTGGAGGCACCTTCAGCAGCTATGCTATCAGCTGGGTGCGACAGGCC CCTGGACAAGGGCTTGAGTGGATGGGA 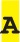 GGATCATCCCTATCTTTGGTACAGCAAACTAC GCACAGAAGTTCCAGGGCAGAGTCACGATTACCGCGGACGAATCCACGAGCACAGCCTAC ATGGAGCTGAGCAGCCTGAGATCTGAGGACACGGCCGTGTATTACTGTGCGAGAGA
IGHV4-31*02 (C198T)	CAGGTGCAGCTGCAGGAGTCGGGCCCAGGACTGGTGAAGCCTTCACAGACCCTGTCCCTC ACCTGTACTGTCTCTGGTGGCTCCATCAGCAGTGGTGGTTACTACTGGAGCTGGATCCGC CAGCACCCAGGGAAGGGCCTGGAGTGGATTGGGTACATCTATTACAGTGGGAGCACCTA 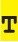 TACAACCCGTCCCTCAAGAGTCGAGTTACCATATCAGTAGACACGTCTAAGAACCAGTTC TCCCTGAAGCTGAGCTCTGTGACTGCCGCGGACACGGCCGTGTATTACTGTGCGAGAGA

*Bold, yellow-highlighted bases indicate single nucleotide polymorphisms from the documented IMGT alleles referenced in the name of the novel allele*.

### gDNA Novel Allele Validation

**Figure 2:**
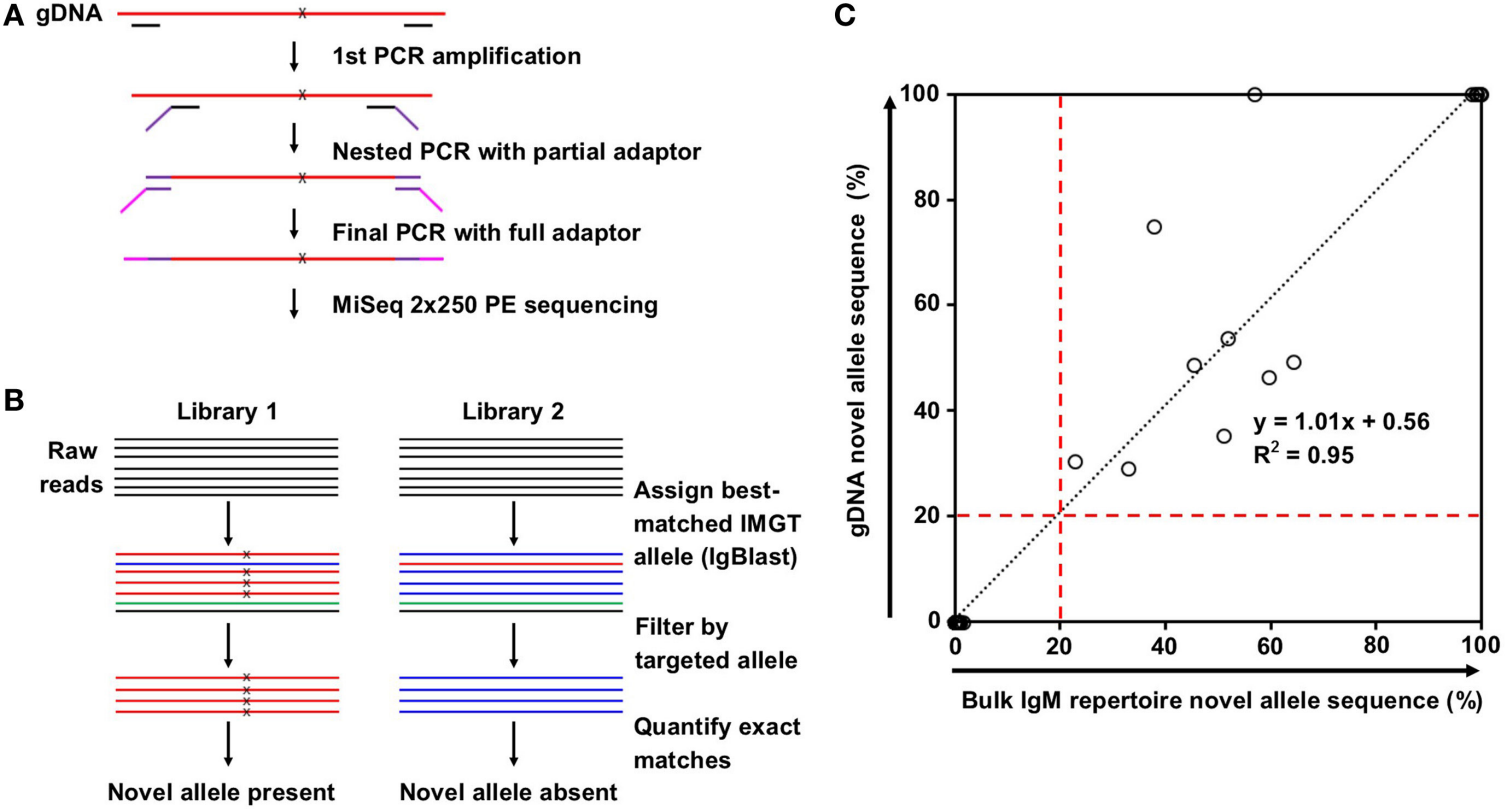
Novel allele validation by targeted genomic DNA (gDNA) sequencing. **(A)** Overview of targeted gDNA amplification and library preparation. x indicates predicted single nucleotide polymorphism (SNP) on novel allele compared to the closest IMGT reference germline allele. **(B)** Overview of genomic DNA (gDNA) sequencing data analysis for the presence (left) and absence (right) of a novel allele resulted from SNP(s). Color indicates best-matched IMGT reference germline allele assignment; x indicates SNP to the closest IMGT reference germline allele. **(C)** Correlation between the percentage of novel allele sequences in bulk IgM repertoire data (%) and the percentage of novel allele sequences in gDNA data (%). Most points are clustered at the origin (*N* = 30) or the top right (*N* = 9). Black dotted line represents the linear regression; red dashed lines indicate the novel allele calling threshold.

Genomic DNA purified from FACS-sorted T cells from the same eight subjects was used to validate the presence of the predicted novel alleles as described in Section “gDNA Sequencing and Reads Processing” and summarized in Figures 2A,B. Due to the high degree of sequence homology among the V genes, a series of filtering steps was applied to eliminate reads that were distant from the putative novel allele or closest IMGT allele. Finally, the number of reads exactly matching the novel and original IMGT sequences were compared. If 20% of these reads matched the novel allele sequence, the subject was deemed positive for the novel allele. All 17 of the positive hits from the bulk repertoire data returned positive hits from the gDNA, and 30 out of 31 negative hits from the bulk repertoire data that were tested in parallel were also negative in the gDNA, with one library failing to amplify (Table 1).

The positive hits in the bulk repertoire data ranged from 22.7 to 99.9% of unique sequences containing the novel mutation, while the negative hits ranged from 0.0 to 1.6% (Figure 2C, *X*-axis). This tight range on the negative hits is consistent with the low rate of mutations expected for IgM antibodies. For the gDNA validation, the positive hits ranged from 29.0 to 100% of filtered reads exactly matching the novel sequence, while the negative hits all failed to detect a single novel allele read (Figure 2C, *Y*-axis). The densely packed clusters at the bottom left and top right of Figure 2C imply that this method is sensitive enough to distinguish between heterozygous and homozygous genotypes, and our threshold of calling a novel allele on both gDNA and repertoire data, which is 20% of reads mapped to either putative allele or its closest IMGT allele, is appropriate.

Another observation that increases confidence in novel germline allele prediction is the detection of the identical novel allele in multiple individuals (12). 5 of the 6 alleles tested were positively validated in two or more subjects (Table 1). The lone allele detected in a single individual, IGHV4-61*01 (C93T_C136G_A138C), is three mismatches away from the nearest IMGT germline allele. None of the gDNA reads for this individual matched the reference allele, while all of the filtered reads exactly matched the predicted novel sequence. Additionally, none of the filtered reads from all seven negative subjects tested in parallel matched the novel sequence.

## Discussion

We developed a remarkably sensitive yet simple method for detecting and validating novel alleles from bulk antibody repertoire sequencing data. This approach requires little specialized bioinformatics analysis and no unique laboratory equipment or reagents. gDNA validation can be performed on DNA purified from as few as 2,000 FACS-sorted non-B cells, maximizing the proportion of the sample that can be utilized for antibody repertoire analysis.

In the era of Big Data and immune repertoire sequencing, researchers attempt to mine meaningful associations out of vast swaths of information. Reliable bioinformatics analysis is highly dependent on the quality data being analyzing. With respect to immune repertoire sequencing, great strides have been made toward mitigating sequencing and PCR errors, but even perfectly accurate sequencing data can result in erroneous SHM calling if there are mismatches between the reference germline alleles and the individual’s true germline sequence. These systemic errors in SHM calling can propagate into faulty conclusions. For example, the SNP in IGHV4-59*01 (T109C) results in an amino acid substitution of tyrosine to histidine in the CDR1. The relative frequency of SHMs that lead to amino acid changes versus those that do not (replacement versus silent mutations) can be used to gauge antigen selection strength—more replacement mutations than expected indicates positive selection while fewer indicates negative selection (17). Mistakenly adding an extra replacement mutation in the CDR to a large portion of the sequences mapped to this allele could give the appearance of affinity maturation and antigen selection when perhaps no such selection took place. Additionally, tracking the evolution of antibody lineages has led to interesting results, particularly in broadly neutralizing HIV antibodies (18). Mistakenly attributing a germline SNP to a SHM could lead to incorrect root assignment or directionality during lineage tree formation, confounding the results. Therefore, it is important to verify germline allele sequences before performing detailed antibody repertoire analysis.

The combination of antibody repertoire and non-B cell gDNA sequencing allowed for advanced insight into the genotypes of the subjects. Novel allele IGHV1-69*01 (G163A) was found to be an exact match with IMGT allele IGHV1-69*07, except IGHV1-69*07 is truncated at both ends. After performing nested PCR, these alleles were indistinguishable. However, in the three subjects predicted to have the novel allele, no unique sequences in the bulk repertoire data mapped to the truncated IGHV1-69*07 allele; instead, they contained the full length IGHV1-69*01 sequence with the G163A SNP.

*IGHV1-69* and *IGHV1-69D* share common alleles that can range from 2 to 4 copies total on a diploid genome, and *IGHV3-30* and *IGHV3-30-5* share common alleles that can range from 0 to 4 copies total on a diploid genome (15). Interestingly, we detected more than 2 alleles in the gDNA of 2 of 8 subjects for *IGHV1-69/1-69D*, consistent with previous studies on *IGHV1-69* CNV in African populations (16), and more than 2 *IGHV3-30/3-30-5* alleles were detected in 3 of 8 subjects (^a^ in Table 1). Conversely, *IGHV4-31* and *IGHV4-61* are associated with deletion events yielding 0 to 4 copies, each (15). *IGHV4-31* was not observed in the repertoire or gDNA of 3 of 8 subjects, and *IGHV4-61* was not observed in the repertoire or gDNA of 2 of 8 subjects (^b^ in Table 1), likely indicating the absence of these genes in these subjects. These results demonstrated the sensitivity of our approach and emphasized the necessity of characterizing individual’s own germline alleles in antibody repertoire sequencing studies in order to accurately count the number of SHMs.

The results were highly consistent with all 17 predicted positive hits and 30 of 31 predicted negative hits confirmed in gDNA. One limitation is that this method will only detect novel alleles that are similar to alleles within the IMGT reference database. Additionally, if a CNV results in more than four alleles present in the diploid genome for a given gene in an individual, then our threshold of a putative SNP call, which is least 20% of unique IgM sequences having the same mismatch at the same position, would not be able to detect the novel allele initially in the antibody repertoire data. However, this is extremely rare based on current knowledge of antibody gene loci (15). In summary, at least 1 novel allele was found in each subject tested, highlighting the need for novel allele detection and correction in antibody repertoire analysis.

## Ethics Statement

The Ethics Committee of the Faculty of Medicine, Pharmacy, and Dentistry at the University of Sciences, Technique, and Technology of Bamako and the Institutional Review Board of the National Institute of Allergy and Infectious Diseases, National Institutes of Health approved the malaria study, from which we obtained frozen PBMCs. Written informed consent was obtained from adult participants and from the parents or guardians of participating children.

## Author Contributions

BW designed and performed research, analyzed and interpreted data, and wrote the manuscript. CH helped perform data analysis. PC and SP provided samples and helped design research. NJ designed research, directed the study, provided funding, and wrote the manuscript.

## Conflict of Interest Statement

NJ is a scientific advisor of ImmuDX LLC. All other authors declare no conflict of interest.

## Footnotes

^1^https://github.com/jstjohn/SeqPrep.
