# Supplementary material for: A Streamlined Approach to Antibody Novel Germline Allele Prediction and Validation
Source: Front Immunol. 2017 Sep 4;8:1072. doi: 10.3389/fimmu.2017.01072 (PMC5591497; doi:10.3389/fimmu.2017.01072)
Supplement: Supplementary file 1 [file Table_1.DOCX]

Supplementary Material

**A streamlined approach to antibody novel germline allele prediction and validation**

**Ben S. Wendel, Chenfeng He, Peter D. Crompton, Susan K. Pierce_,_ Ning Jiang***

*** Correspondence:** Ning Jiang: jiang@austin.utexas.edu

**Supplementary Table 1.** gDNA validation primer list. Sequences in red indicate common partial Illumina adaptors; NNNNNN in blue indicates fixed library indexes used to pool multiple libraries into a single run.

| **Target** | **Forward Primer Sequence** | **Reverse Primer Sequence** | **PCR Stage** |
| --- | --- | --- | --- |
| IGHV1-8*02 (G234T) | GGGCTGAGGTGAAGAAGC | CCTCTCGCACAGTAATACACG | 1st PCR |
| IGHV3-30*02 (T201C) | GTGCAGCTGGTGGAGTC | CTTTCGCACAGTAATACACAGC |  |
| IGHV4-61*01 (C93T_C136G_A138C) | GACTGGTGAAGCCTTCGG | TCTCTCGCACAGTAATACACG |  |
| IGHV4-59*01 (T109C) | GCCCAGGACTGGTGAAG | TCTCTCGCACAGTAATACACG |  |
| IGHV1-69*01 (G163A) | GGGCTGAGGTGAAGAAGC | TCTCTCGCACAGTAATACACG |  |
| IGHV4-31*02 (C198T) | GCCCAGGACTGGTGAAG | TCTCTCGCACAGTAATACACG |  |
| IGHV1-8*02 (G234T) | GACGTGTGCTCTTCCGATCT  GGGCCTCAGTGAAGGTCT | ACACTCTTTCCCTACACGACGCTCTTCCGATCT TCAGATCTCAGGCTGCTCA | Nested PCR |
| IGHV3-30*02 (T201C) | GACGTGTGCTCTTCCGATCT  GTCCCTGAGACTCTCCTGT | ACACTCTTTCCCTACACGACGCTCTTCCGATCT AGCTCTCAGGCTGTTCATTT |  |
| IGHV4-61*01 (C93T_C136G_A138C) | GACGTGTGCTCTTCCGATCT  CTCACCTGCACTGTCTCTG | ACACTCTTTCCCTACACGACGCTCTTCCGATCT GTCACAGAGCTCAGCTTCA |  |
| IGHV4-59*01 (T109C) | GACGTGTGCTCTTCCGATCT  GACCCTGTCCCTCACCT | ACACTCTTTCCCTACACGACGCTCTTCCGATCT TCACAGAGCTCAGCTTCA |  |
| IGHV1-69*01 (G163A) | GACGTGTGCTCTTCCGATCT  TCCTCGGTGAAGGTCTCC | ACACTCTTTCCCTACACGACGCTCTTCCGATCT GCTGCTCAGCTCCATGT |  |
| IGHV4-31*02 (C198T) | GACGTGTGCTCTTCCGATCT  CCCTGTCCCTCACCTGTA | ACACTCTTTCCCTACACGACGCTCTTCCGATCT GTCACAGAGCTCAGCTTCA |  |
| Illumina Adaptors | CAAGCAGAAGACGGCATACGAGATAANNNNNN GTGACTGGAGTTCAGACGTGTGCTCTTCCGATCT | AATGATACGGCGACCACCGAGATCT ACACTCTTTCCCTACACGAC | Adaptor Extension |
